# Multicenter Analysis of Valganciclovir Prophylaxis in Pediatric Solid Organ Transplant Recipients

**DOI:** 10.1093/ofid/ofae353

**Published:** 2024-07-01

**Authors:** Marc Foca, Salih Demirhan, Flor M Munoz, Kristen G Valencia Deray, Claire E Bocchini, Tanvi S Sharma, Gilad Sherman, William J Muller, Taylor Heald-Sargent, Lara Danziger-Isakov, Samantha Blum, Juri Boguniewicz, Samantha Bacon, Tuhina Joseph, Jodi Smith, Monica I Ardura, Yin Su, Gabriela M Maron, Jose Ferrolino, Betsy C Herold

**Affiliations:** Division of Infectious Diseases, Department of Pediatrics, Children's Hospital at Montefiore, Albert Einstein College of Medicine, Bronx, New York, USA; Division of Infectious Diseases, Department of Pediatrics, Children's Hospital at Montefiore, Albert Einstein College of Medicine, Bronx, New York, USA; Division of Infectious Diseases, Department of Pediatrics, Texas Children's Hospital, Baylor College of Medicine, Houston, Texas, USA; Division of Infectious Diseases, Department of Pediatrics, Texas Children's Hospital, Baylor College of Medicine, Houston, Texas, USA; Division of Infectious Diseases, Department of Pediatrics, Texas Children's Hospital, Baylor College of Medicine, Houston, Texas, USA; Division of Infectious Diseases, Department of Pediatrics, Boston Children's Hospital, Harvard Medical School, Boston, Massachusetts, USA; Division of Infectious Diseases, Department of Pediatrics, Boston Children's Hospital, Harvard Medical School, Boston, Massachusetts, USA; Division of Infectious Diseases, Department of Pediatrics, Ann & Robert H. Lurie Children's Hospital, Northwestern University Feinberg School of Medicine, Chicago, Illinois, USA; Division of Infectious Diseases, Department of Pediatrics, Ann & Robert H. Lurie Children's Hospital, Northwestern University Feinberg School of Medicine, Chicago, Illinois, USA; Division of Infectious Diseases, Department of Pediatrics, Cincinnati Children's Hospital Medical Center, University of Cincinnati, Cincinnati, Ohio, USA; Division of Infectious Diseases, Department of Pediatrics, Cincinnati Children's Hospital Medical Center, University of Cincinnati, Cincinnati, Ohio, USA; Division of Infectious Diseases, Department of Pediatrics, Children's Hospital Colorado, University of Colorado School of Medicine, Aurora, Colorado, USA; Division of Infectious Diseases, Department of Pediatrics, Children's Hospital Colorado, University of Colorado School of Medicine, Aurora, Colorado, USA; Division of Infectious Diseases, Department of Pediatrics, Boston Children's Hospital, Harvard Medical School, Boston, Massachusetts, USA; Division of Nephrology, Department of Pediatrics, Seattle Children's Hospital, University of Washington Medical School, Seattle, Washington, USA; Division of Infectious Diseases and Host Defense, Department of Pediatrics, Nationwide Children's Hospital, The Ohio State University, Columbus, Ohio, USA; Department of Infectious Diseases, St Jude Children's Research Hospital, Memphis, Tennessee, USA; Department of Infectious Diseases, St Jude Children's Research Hospital, Memphis, Tennessee, USA; Department of Infectious Diseases, St Jude Children's Research Hospital, Memphis, Tennessee, USA; Division of Infectious Diseases, Department of Pediatrics, Children's Hospital at Montefiore, Albert Einstein College of Medicine, Bronx, New York, USA

**Keywords:** Pediatrics, Solid Organ Transplantion, Cytomegalovirus (CMV)

## Abstract

**Background:**

Valganciclovir is the only approved antiviral for cytomegalovirus (CMV) prevention in pediatric solid organ transplantation (SOT). Additional approaches may be needed to improve outcomes.

**Methods:**

A multicenter retrospective study from 2016 to 2019 was conducted of pediatric SOT recipients in whom at least 3 months of valganciclovir prophylaxis was planned. Episodes of CMV DNA in blood (DNAemia), CMV disease, drug-related toxicities, as well as other infections in the first year posttransplant and demographic and clinical data were collected. CMV DNAemia in the first year after prophylaxis or during prophylaxis (breakthrough) was analyzed by multivariate hazard models.

**Results:**

Among the 749 patients enrolled, 131 (17.5%) had CMV DNAemia at any time in the first year; 85 (11.4%) had breakthrough DNAemia, and 46 (6.1%) had DNAemia after prophylaxis. CMV disease occurred in 30 (4%). In a multivariate model, liver transplantation compared to kidney or heart, intermediate or high risk based on donor/recipient serologies, neutropenia, and valganciclovir dose modifications attributed to toxicity were associated with increased risk of total and/or breakthrough DNAemia. Bacteremia was also associated with increased hazard ratio for CMV DNAemia. In a separate multivariate analysis, rejection occurred more often in those with breakthrough CMV DNAemia (*P* = .002); liver transplants, specifically, had increased rejection if CMV DNAemia occurred in the first year (*P* = .004). These associations may be bidirectional as rejection may contribute to infection risk.

**Conclusions:**

CMV DNAemia in the first year posttransplantation occurs despite valganciclovir prophylaxis and is associated with medication toxicity, bacteremia, and rejection. Pediatric studies of newer antivirals, especially in higher-risk subpopulations, appear to be warranted.

## Teaser

Valganciclovir prophylaxis has reduced the incidence of cytomegalovirus (CMV) disease in children post–solid organ transplantation, but the incidence of breakthrough CMV reactivation during prophylaxis and in the first year posttransplantation highlights the need for studies of new agents for pediatric populations.

The introduction of valganciclovir (VGCV) for prophylaxis or preemptive treatment of cytomegalovirus (CMV) infection in pediatric solid organ transplantation (SOT) has simplified management over the past 2 decades. Prior to the widespread availability of this agent, transplant programs relied on oral ganciclovir with a bioavailability of only 6%–10% [1] or valacyclovir, which required high doses, reflecting limited phosphorylation of acyclovir by CMV UL97 [2]. Both oral ganciclovir and valacyclovir also required administration 3–4 times daily. VGCV provided a once-daily prophylactic option that had similar efficacy to oral ganciclovir [3, 4] and a twice-daily treatment option with pharmacokinetics similar to intravenous ganciclovir [5].

Despite these improvements, there remain significant clinical questions concerning VGCV including relative safety and efficacy of universal prophylaxis versus preemptive therapy [6, 7], optimal dosing strategies, and drug-associated neutropenia or lymphopenia that may lead to premature discontinuation. Prior studies have suggested an association between CMV and graft rejection or loss in pediatric and adult cohorts [8–11], as well as increased rates of other infections, most notably *Pneumocystis jirovecii* pneumonia (PJP), in adult renal transplantation [12]. Moreover, neutropenia and/or lymphopenia, which were observed more often in patients who developed breakthrough CMV DNAemia, may also predispose to other infections.

To assess the need for alternative approaches, we conducted a retrospective multicenter study in children in whom at least 3 months of VGCV prophylaxis was planned. The primary outcomes assessed were incidence of CMV infection, defined as the detection of viral DNA in the blood by quantitative plasma polymerase chain reaction (PCR) (DNAemia), or disease either during VGCV prophylaxis (breakthrough) and/or after completion of prophylaxis within the first year posttransplantation. We also examined potential risk factors associated with CMV as well as rates of rejection, other infections, and 1-year survival in patients who did or did not develop CMV DNAemia.

## METHODS

### Study Design

We conducted a multicenter retrospective study of VGCV prophylaxis in pediatric SOT patients at 8 US sites who participate in the Pediatric Infectious Diseases Transplant Research Network (Supplementary Table 1). Each site obtained ethical approval from its institutional review board (IRB). SOT recipients <20 years of age who were transplanted for the first time between January 2016 and December 2019, were anticipated to receive VGCV prophylaxis for at least 3 months, and for whom 1-year posttransplant outcomes were available were included in the study. The REDCap electronic data tool hosted by St Jude Children's Research Hospital was used to manage the data for each patient, which were extracted using the electronic medical record [14, 15].

The VGCV duration and dosing were based on internal protocols at each center as previously described (Supplementary Table 2) [13]. Similarly, the induction and maintenance immunosuppressive agents and the use of trimethoprim-sulfamethoxazole for PJP prophylaxis were at the discretion of each center and individual transplant program.

### Patient Consent Statement

Local IRB approval with waiver of consent was obtained at all institutions: Albert Einstein, Texas Children's, Boston Children's, Lurie Children's, Cincinnati Children's, Children's Hospital of Colorado, Seattle Children's, Nationwide Children's Hospital, and St Jude Research Hospital.

### Outcome Measures and Definitions

The primary viral outcome measures included CMV DNAemia or disease while receiving VGCV prophylaxis (breakthrough) or after completion of prophylaxis within the first year after transplant. Documented tissue invasive CMV infection or CMV DNAemia with clinical signs such as fever, hepatitis, or colitis was defined as CMV disease. Donor (D) and recipient (R) anti-CMV immunoglobulin G serostatus was used to categorize CMV risk groups: low risk (D^−^/R^−^), intermediate risk (D^+^/R^+^ or D^−^/R^+^), or high risk (D^+^/R^−^). Cytopenia and severe cytopenia (neutropenia and lymphopenia) were defined as absolute cell counts <1000 cells/μL and <500 cells/μL, respectively. Those cytopenias that occurred within the first 15 days posttransplantation were excluded as they could represent the effects of transplantation induction therapy. Rejection was recorded as any episode within the first year after transplantation and was defined by institutional protocol. No date or relative timing of rejection event compared to breakthrough CMV DNAemia was recorded.

### Laboratory Assays

During the study period, all centers used quantitative PCR to detect CMV DNAemia and recorded results based on standardized World Health Organization international units (IU) [16, 17]. The sample type (eg, whole blood or plasma), assay, and testing frequency were at the discretion of each study site. Complete blood count results were collected at approximately days 0, 30, 90, 180, and 365 post-SOT, and during episodes of VGCV-associated toxicities or CMV DNAemia.

### Statistical Analysis

Stata software, version 17.0 (StataCorp LLC) was used for performing all statistical analyses. Both visual (histogram) and statistical assessment (skewness and kurtosis tests) was used to determine the distribution of continuous variables. Categorical variables were expressed as numbers (percentages), whereas continuous variables were expressed as the median (interquartile range [IQR], 25%–75%). The χ^2^ and Mann-Whitney *U* tests were used for the bivariate analysis of categorical variables and continuous variables, respectively. Adjusted and unadjusted Cox regression models were generated for each primary outcome except for CMV disease due to insufficient event numbers. Dosing strategy, transplanted organ, age, CMV risk status, VGCV therapy duration, and other potential confounders with *P* < .1 in the binary analysis were included in the models. Backward elimination, starting with the independent variable with the highest *P* value, was performed. Independent variables were removed from the model one at a time and were added back to the multivariable regression models to evaluate if they changed the coefficient in either direction by 10% or more. Separate logistic regression models were created for rejection outcome adjusted for CMV DNAemia and transplanted organ type. Variables were assessed for first-order interaction on the association between VGCV dosing method and outcome. A *P* value <.05 was considered statistically significant.

## RESULTS

### Clinical and Demographic Features Associated With CMV DNAemia

There were 749 pediatric transplant patients enrolled in the study, and 131 (17.5%) developed CMV DNAemia at any time in the first year after transplantation. This included 85 (11.4% of the total cohort) who developed breakthrough CMV DNAemia (Table 1). Among these 85 patients, there were 107 breakthrough CMV episodes. The median duration from transplantation to CMV infection was 112 (IQR, 30–230) days. Twenty-seven episodes occurred in the first 30 days, 21 occurred between 31 and 90 days, 27 between 91 and 180 days, and 32 between 181 and 365 days. Seventy-two patients had a single episode of breakthrough infection, 8 patients had 2 episodes, 3 patients had 3 episodes, and 1 patient each had 4 and 6 episodes over the course of the study. Among these breakthrough events, 74 of 107 (69%) were treated. The valganciclovir dose was increased from prophylaxis to treatment in 48.6% (52/107), ganciclovir was started in 27 (25%), and foscarnet was prescribed in 1 episode. More than 1 therapeutic modality could have been used during an episode.

**Table 1.** Clinical and Demographic Features of the Study Population Comparing Patients Who Did or Did Not Develop Cytomegalovirus Infection or Disease

Characteristic	Overall (n = 749)	No DNAemia (n = 618)	Breakthrough DNAemia (n = 85)	Post-PPX DNAemia (n = 46)	Overall *P* Value	p1^a^	p2^b^	p3^c^
Demographics								
Age, y, median (IQR)	8.4 (2.3–13.8)	9.0 (2.4–13.7)	5.5 (1.4–14.1)	7.6 (3.4–12.3)	.2	.1	.9	.2
Sex, female	339 (45.3)	277 (44.8)	42 (49.4)	20 (43.5)	.7	.4	.9	.5
Race					.2	.1	.4	.1
White	264 (67.5)	212 (69.3)	28 (54.9)	24 (70.6)				
Black	53 (13.6)	39 (12.8)	7 (13.7)	7 (20.6)				
Asian	20 (5.1)	15 (4.9)	4 (7.8)	1 (2.9)				
Other/declined	54 (13.8)	40 (13.1)	12 (23.5)	2 (5.9)				
Ethnicity					.2	.7	.09	.2
Hispanic	193 (26.1)	156 (25.6)	20 (23.8)	17 (37.0)				
Clinical features								
Transplanted organ					<.001	<.001	.07	.3
Kidney	294 (39.3)	269 (43.5)	14 (16.5)	11 (23.9)				
Liver	227 (30.3)	169 (27.4)	39 (45.9)	19 (41.3)				
Heart	169 (22.6)	136 (22.0)	19 (22.4)	14 (30.4)				
Lung	40 (5.3)	30 (4.9)	9 (10.6)	1 (2.2)				
Other	19 (2.5)	14 (2.3)	4 (4.7)	1 (2.2)				
CMV risk group					<.001	<.001	.002	.2
High-risk (D^+^/R^−^)	316 (42.7)	245 (40.1)	49 (59.0)	22 (47.8)				
Intermediate-risk (D^+^/R^+^ or D^−^/R^+^)	291 (39.3)	235 (38.5)	32 (38.6)	24 (52.2)				
Low-risk (D^−^/R^−^)	133 (18.0)	131 (21.4)	2 (2.4)	0				
Duration of VGCV PPX, mo, median (IQR)	180 (90–365)	180 (90–365)	365 (180–365)	90 (90–180)	<.001	<.001	<.001	<.001
CMV IVIG PPX	104 (14.1)	79 (13.0)	13 (15.7)	12 (26.1)	.04	.5	.01	.2
TMP-SMX PPX	584 (78.0)	470 (76.1)	75 (88.2)	39 (84.8)	.02	.01	.2	.6
CMV disease	30 (4.0)	0	17 (20.0)	13 (28.3)	<.001	<.001	<.001	.3
CMV treatment (disease or DNAemia)	93 (12.4)	0	63 (74.1)	30 (65.2)	<.001	<.001	<.001	.3

Data are presented as No. (%) unless otherwise indicated.

Abbreviations: CMV, cytomegalovirus; D^–^, donor negative; D^+^, donor positive; IQR, interquartile range; IVIG, intravenous immunoglobulin; PPX, prophylaxis; R^–^, recipient negative; R^+^, recipient positive; TMP-SMX, trimethoprim-sulfamethoxazole; VGCV, valganciclovir.

^a^No DNAemia vs breakthrough DNAemia.

^b^No DNAemia vs postprophylaxis DNAemia.

^c^Breakthrough DNAemia vs postprophylaxis DNAemia.

Forty-six patients (6.1%) developed 53 episodes of CMV DNAemia after completion of prophylaxis (Table 1). The median time from VGCV discontinuation to CMV infection was 83 (IQR, 42–105) days. Those who developed postprophylaxis DNAemia were more likely to have had a shorter duration of daily VGCV (90 [IQR, 90–180] days) than either those with no DNAemia (180 [IQR, 90–365] days) or breakthrough infection (365 [180–365] days) (*P* < .001 for both).

There were no significant differences in age, sex, race, or ethnicity comparing those who did or did not develop CMV DNAemia at any time in the first year after transplantation (Table 1). As expected, CMV DNAemia at any time was more common in serologically high-risk (CMV D^+^/R^−^) or intermediate-risk (CMV D^+^/R^+^ or CMV D^−^/R^+^) transplants and almost never detected in the low-risk (CMV D^−^/R^−^) dyads. There were no significant differences in induction or maintenance immunosuppressive therapy based on the primary outcome, with the exception that alemtuzumab, while only used 9.5% of the time for induction, was used more frequently in those who did not develop CMV (Supplementary Table 3). However, this likely reflects its use primarily among low-risk renal transplant patients at 1 center.

CMV disease occurred in only 30 patients (4% of the total cohort), including 17 (20%) of those with breakthrough and 13 (28%) of those with postprophylaxis DNAemia. Treatment with intravenous ganciclovir or increased VGCV dosage was initiated in 93 of the 131 patients with CMV DNAemia, including 63 of 85 (74.1%) of those with breakthrough and 30 of 46 (65.2%) of those who developed CMV after completing prophylaxis. The threshold for initiating treatment was at the discretion of the treating physician, and these data was not collected. However, peak plasma PCR values for those treated were 3900 (IQR, 1300–18 307) IU/mL versus 898 (IQR, 376–1507) IU/mL for those who were not treated (*P* < .001).

Impact of Organ Type on Risk for CMV DNAemia

There were 294 kidney, 227 liver, and 160 heart transplant recipients in the cohort as well as a smaller number of lung (n = 40) and other (intestinal or multiorgan) (n = 19) transplant recipients. Notably, liver transplant recipients, compared with kidney recipients, had a greater number of patients with breakthrough CMV DNAemia (*P* < .001) and treatment for breakthrough CMV (*P* < .001) (Table 2). The percentages of each these were comparable in liver and lung transplant recipients. Although there were significant differences in induction and maintenance immunosuppression regimens by transplanted organ (Supplementary Table 4), these differences were not associated with breakthrough CMV. For example, liver transplant recipients received less overall immunosuppression (anti-thymocyte globulin or alemtuzumab) compared to kidney transplant recipients (Supplementary Table 4, *P* < .001), yet kidney recipients were least likely to develop breakthrough CMV DNAemia (4.8%) or postprophylaxis DNAemia (3.9%). However, when they did have detectable CMV, the peak values were greater than those in the other organ groups, although the range was large.

**Table 2.** Comparison of Cytomegalovirus Infections Among Patients Who Received Different Organs

CMV Infection and Disease	Overall (n = 749)	Kidney (n = 294)	Liver (n = 227)	Heart (n = 169)	Lung (n = 40)	Overall *P* Value
Breakthrough CMV DNAemia	85 (11.4)	14 (4.8)	39 (17.2)	19 (11.2)	9 (22.5)	<.001^a^
CMV disease	17 (2.3)	6 (2.0)	7 (3.1)	3 (1.8)	1 (2.5)	.8
CMV treatment	63 (8.4)	12 (4.1)	30 (13.2)	11 (6.5)	7 (17.5)	<.001^b^
Postprophylaxis CMV DNAemia	46 (6.9)	11 (3.9)	19 (10.1)	14 (9.3)	1 (6.7)	.07
CMV disease	13 (1.7)	2 (0.7)	6 (2.6)	3 (1.8)	1 (2.5)	.3
CMV treatment	30 (4.0)	9 (3.1)	12 (5.3)	7 (4.1)	1 (5.3)	.7
Peak CMV PCR, IU/mL, median (IQR)	2214 (805–11 674)	7943 (1507–45 348)	1617 (839–6440)	2214 (1014–12 322)	1709 (598–8519)	.04^c^

Data are presented as No. (%) unless otherwise indicated.

Abbreviations: CMV, cytomegalovirus; IQR, interquartile range; PCR, polymerase chain reaction.

^a^Kidney vs liver: <.001; kidney vs heart: .009; kidney vs lung: <.001; liver vs heart: .1; liver vs lung: .4; heart vs lung: .06.

^b^Kidney vs liver: <.001; kidney vs heart: .2; kidney vs lung: .007; liver vs heart: .03; liver vs lung: .8; heart vs lung: .1.

^c^Kidney vs liver: .01; kidney vs heart: .2; kidney vs lung: .1; liver vs heart: .3; liver vs lung: .9; heart vs lung: .6.

VGCV Is Associated With Increased Frequency of Neutropenia and Lymphopenia

Neutropenia and lymphopenia were documented in 35.9% and 30.4% of patients within the first year after transplantation, respectively (Table 3). These cytopenias occurred more often in those who developed breakthrough CMV compared to those who never developed CMV DNAemia (neutropenia: 48.2% vs 26.5%, *P* < .001; lymphopenia: 40.0% vs 23.0%, *P* = .001). There was no difference in the percentage of patients who received granulocyte colony-stimulating factor (filgrastim). The VGCV dose was reduced because of toxicity attributed by the treating physician to the drug more often in those who subsequently developed breakthrough CMV DNAemia compared to those who did not (22.4% vs 9.2%, *P* < .001; Table 3). Although it is difficult to infer a causal relationship, 18 of 19 breakthrough CMV episodes that occurred with a VGCV modification followed the dose adjustment.

**Table 3.** Episodes of Neutropenia and Lymphopenia

VGCV Toxicity	Overall (n = 749)	No DNAemia (n = 618)	Breakthrough (n = 85)	Post-PPX (n = 46)	Overall *P* Value	p1^a^	p2^b^	p3^c^
Neutropenia^d^								
Within 1 y posttransplantation	269 (35.9)	200 (32.4)	48 (56.5)	21 (45.7)	<.001	<.001	.07	.2
While on valganciclovir	220 (29.4)	164 (26.5)	41 (48.2)	15 (32.6)	<.001	<.001	.4	.08
Severe neutropenia	165 (22.0)	126 (20.4)	28 (32.9)	11 (23.9)	.03	.009	.6	.3
Nadir ANC, cells/μL, median (IQR)	400 (190–650)	400 (199–620)	400 (140–750)	331 (57–650)	.8	.7	.6	.5
Filgrastim for neutropenia	102 (13.6)	82 (13.3)	13 (15.3)	7 (15.2)	.8	.6	.7	.9
Lymphopenia^e^								
Within 1 y posttransplantation	228 (30.4)	173 (28.0)	37 (43.5)	18 (39.1)	.006	.003	.1	.6
While on valganciclovir	187 (25.0)	142 (23.0)	34 (40.0)	11 (23.9)	.003	.001	.9	.06
Severe lymphopenia	107 (14.3)	81 (13.1)	17 (20.0)	9 (19.6)	.1	.09	.2	.9
Nadir ALC, cells/μL, median (IQR)	520 (370–798)	510 (370–800)	598 (400–790)	515 (320–700)	.6	.5	.7	.5
VGCV dose reduction or premature discontinuation	78 (10.4)	57 (9.2)	19 (22.4)	2 (4.4)	<.001	<.001	.3	.007

Data are presented as No. (%) unless otherwise indicated.

Abbreviations: ALC, absolute lymphocyte count; ANC, absolute neutrophil count; IQR, interquartile range; PPX, prophylaxis; VGCV, valganciclovir.

^a^No DNAemia vs breakthrough DNAemia.

^b^No DNAemia vs postprophylaxis DNAemia.

^c^Breakthrough DNAemia vs postprophylaxis DNAemia.

^d^Neutropenia: ANC <1000 cells/µL; severe neutropenia: ANC <500 cells/µL.

^e^Lymphopenia: ALC <1000 cells/µL; severe lymphopenia: ALC <500 cells/µL.

CMV DNAemia Is Associated With Increased Episodes of Rejection and Other Infections

Rejection within the first year of transplantation was documented in 167 (22.5%) of the total cohort and was more common among those who experienced breakthrough CMV DNAemia compared to those who did not (35.3% vs 20.6%, *P* = .002) but did not differ significantly between those who developed postprophylaxis CMV versus no CMV (Table 4).

**Table 4.** Transplant Outcomes and Other infections

Outcome	Overall (n = 749)	No DNAemia (n = 618)	Breakthrough (n = 85)	Post-PPX (n = 46)	Overall *P* Value	p1^a^	p2^b^	p3^c^
Rejection	167 (22.5)	126 (20.6)	30 (35.3)	11 (23.9)	.009	.002	.6	.2
Bacteremia	68 (9.1)	48 (7.8)	16 (18.8)	4 (8.7)	.004	.001	.8	.1
Urinary tract infection	97 (13.0)	83 (13.4)	10 (11.8)	4 (8.7)	.6	.7	.4	.6
*Clostridioides difficile*	29 (3.9)	22 (3.6)	5 (5.9)	2 (4.4)	.6	.3	.8	.7
EBV viremia	206 (27.5)	159 (25.7)	32 (37.7)	15 (32.6)	.05	.02	.3	.6
Adenovirus viremia	21 (2.8)	14 (2.3)	7 (8.2)	0	.004	.002	.3	.04
1-y survival	734 (98.0)	608 (98.4)	84 (98.8)	42 (91.3)	.004	.8	.001	.03

Data are presented as No. (%) unless otherwise indicated.

Abbreviations: EBV, Epstein-Barr virus; PPX, prophylaxis.

^a^No DNAemia vs breakthrough DNAemia.

^b^No DNAemia vs postprophylaxis DNAemia.

^c^Breakthrough DNAemia vs postprophylaxis DNAemia.

Almost 50% of patients developed either a bacterial or viral infection (other than CMV) posttransplantation (Table 4). Fungal infections were infrequent (n = 9 total) and there were no cases of PJP in the cohort. Bacteremia was more common in those with breakthrough CMV DNAemia compared to no CMV (18.8% vs 7.8%, *P* = .001). In 16 patients with both breakthrough CMV and bacteremia, 7 had breakthrough CMV first, 7 had bacteremia first, and 2 were not classified. Notably, patients who had bacteremia were more likely to be neutropenic while on valganciclovir (28/68 [41.2%]) compared to those who did not develop bacteremia (192/681 [28.2%]) (*P* = .03). Similarly, adenovirus and Epstein-Barr virus (EBV) DNAemia were also more common in those with breakthrough CMV compared to no CMV (*P* = .002 and *P* = .02, respectively) (Table 4). Seven patients had both breakthrough CMV and adenovirus, of whom 6 (86%) had breakthrough before adenovirus infection. Similarly, 32 patients had both EBV DNAemia and breakthrough CMV, and 20 of these (62.5%) had breakthrough before EBV.

Infections differed when comparing the organ transplanted. Liver transplant recipients experienced 41.6% of all viral infections, including 44.7% of EBV episodes and 57% of adenovirus infections (Supplementary Table 5). Urinary tract infections were more common in kidney transplant recipients, and BK viremia was only detected in that subgroup (Supplementary Table 5). Overall, 1-year survival was 98% and did not differ comparing those breakthrough versus no CMV DNAemia but was lower (91.3%) in those who experienced postprophylaxis CMV compared to the other groups (*P* = .001).

Risk Factors Associated With Breakthrough CMV and Rejection

**Figure 1.**
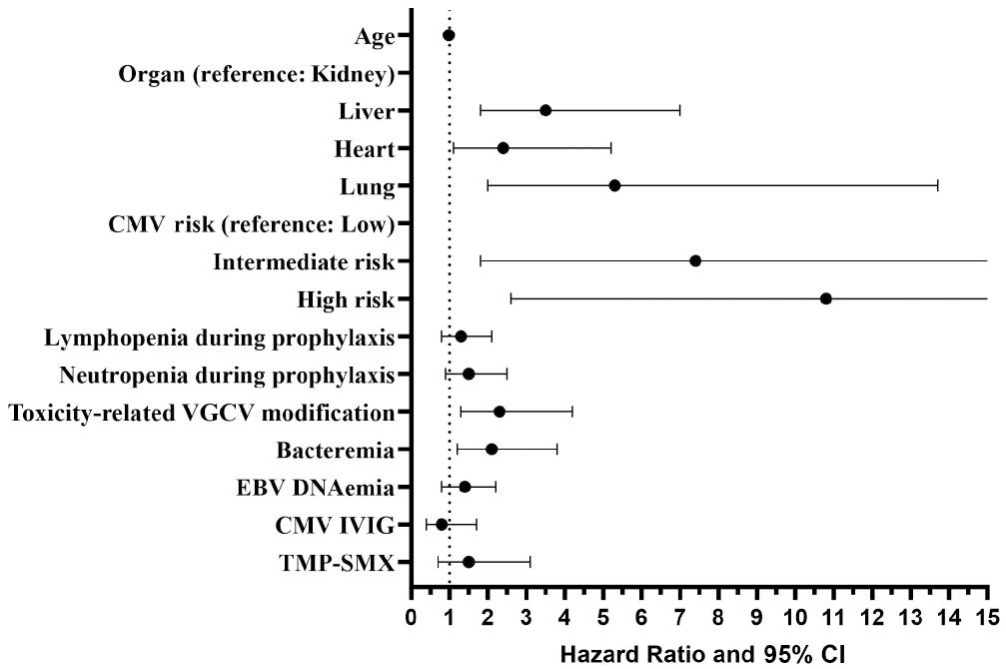
Cox regression model for breakthrough cytomegalovirus DNAemia. Abbreviations: CI, confidence interval; CMV, cytomegalovirus; EBV, Epstein-Barr virus; IVIG, intravenous immunoglobulin; TMP-SMX, trimethoprim-sulfamethoxazole; VGCV, valganciclovir.

Adjusted and unadjusted Cox regression models were generated to identify risk factors associated with breakthrough DNAemia (Figure 1 and Supplementary Table 6), and a separate logistic regression model was generated to identify risk factors associated with rejection (Supplementary Table 7). Using kidney transplant recipients as the reference group and after adjusting for variables that were significant in the univariate analysis, liver (hazard ratio [HR], 3.5 [95% confidence interval [CI], 1.7–7.0]), lung (HR, 5.3 [95% CI, 2.0–13.7]) and heart (HR, 2.4 [95% CI, 1.1–5.2]) transplants had a higher risk of breakthrough CMV DNAemia. High (HR, 10.8 [95% CI, 2.6–45.1]) and intermediate (HR, 7.4 [95% CI, 1.8–31.4]) CMV risk as determined by serologies obtained prior to transplantation was also significantly associated with breakthrough infection. VGCV dose adjustment (HR, 2.3 [95% CI, 1.3–4.2] and bacteremia (HR, 2.1 [95% CI, 1.2–3.8]) were also associated with increased risk for CMV DNAemia. In an adjusted logistic regression model, rejection was associated with the transplanted organ (liver odds ratio [OR], 2.4, *P* < .001; heart OR, 2.9, *P* <.001) and with breakthrough CMV DNAemia (OR, 1.9, *P* = .01) (Supplementary Table 7). In a subanalysis by transplanted organ, the likelihood of experiencing rejection was significantly associated with breakthrough CMV DNAemia in liver transplant recipients (OR, 2.9, *P* = .004) (Supplementary Table 7).

## DISCUSSION

This multicenter study of 749 pediatric SOT patients who initiated a planned minimum of 3 months of VGCV prophylaxis for CMV at 8 geographically distinct US centers documented an incidence of breakthrough CMV DNAemia of 11.4% and any CMV DNAemia within the first year posttransplant of 17.5%. These rates are consistent with several single-center studies, which documented incidences of breakthrough CMV in 5%–53%, and CMV DNAemia at any time in the first year between 11% and 27% of patients [18–22]. The incidence of CMV disease was relatively low in the current multicenter study (4%) as well as in the prior single-center studies (1%–6%), indicating that VGCV prophylaxis is more effective in reducing disease than in preventing breakthrough DNAemia.

VGCV was first studied for the treatment of CMV retinitis in patients with advanced human immunodeficiency virus disease [23, 24]. It was subsequently evaluated in adult SOT patients for prophylaxis and was found to be noninferior to oral and intravenous ganciclovir [3]. There were signals, however, that in liver transplantation, there might be an increased risk of CMV disease in patients receiving VGCV prophylaxis [25]. This was confirmed in a meta-analysis of 9 studies (N = 1831 adult patients), which found an increased risk (4.5-fold) of tissue-invasive CMV in liver transplant recipients who received VGCV compared to other prophylactic agents [25]. However, VGCV prophylaxis was widely adopted because it could be administered orally once a day, was well tolerated, and was overall efficacious. Consensus guidelines have continued to recommend VGCV for CMV prophylaxis, although the optimal duration of prophylaxis and the relative benefits of universal versus targeted or preemptive prophylaxis remains controversial. Despite a lack of large-scale pharmacokinetic or efficacy data in pediatrics, VGCV was similarly adopted for CMV prophylaxis in pediatric SOT settings.

We confirmed the previously established risk factor of donor and recipient CMV serostatus but also found that liver transplant was associated with a significantly greater risk of breakthrough CMV compared to heart or kidney transplant; the reasons for this require further study. The higher incidence observed is not likely attributable to differences in immunosuppressive therapy as liver transplant recipients were less likely than kidney transplant recipients to have received anti-thymocyte globulin or alemtuzumab during induction. These agents promote CMV reactivation presumably because of their prolonged and global immunosuppressive effects. In addition, fever that occurs after administration of these agents triggers the release of tumor necrosis factor, which may stimulate CMV replication through activation of nuclear factor κB [26]. CMV establishes latency in myeloid cells and thus, patients who receive a liver from a CMV-positive donor may be exposed to a greater viral burden compared to recipients of other organs as the number of myeloid cells is higher. This may also contribute to the higher rates of EBV DNAemia we observed in liver compared to the other organ transplant groups as EBV is also latent in immune cells (primarily B cells). Viral reactivation is triggered by inflammatory cytokines and immune cell activation, which are features of engraftment and tissue repair [27]. We speculate that exposure to greater viral reservoir and suppression of antiviral cytolytic T cells needed to maintain latency [28], coupled with the inflammatory response to the graft, converge to increase the risk of CMV (and EBV) reactivation. However, we cannot exclude the possibility that more frequent monitoring contributed to the observed higher incidence in liver transplant recipients.

Both neutropenia and lymphopenia were relatively common in the first year posttransplantation, but only neutropenia was potentially associated with breakthrough CMV DNAemia in the multivariate analysis. Whether the neutropenia itself contributed to the risk for CMV reactivation, provided a biomarker of immunologic changes that promote CMV, or was presumed to represent a drug-associated toxicity resulting in dose reduction cannot be disentangled. However, modification of the VGCV dose based on concerns for drug-associated toxicities was independently associated with breakthrough CMV. This finding is consistent with a single-center retrospective study, which also found that VGCV dose reduction was an independent risk factor for breakthrough CMV DNAemia in pediatric SOT recipients [21]. The need to adjust the dose for presumed drug-associated adverse events is mitigated, in part, when weight-based rather than manufacturer-recommended body surface area (BSA) dosing is used. In a recently published study involving a subset of the current cohort, we found that CMV DNAemia (breakthrough or at any time in the first year) occurred no more often with weight-based dosing whereas neutropenia and lymphopenia leading to dosing modifications were documented more frequently in those receiving BSA-based dosing [13]. A single-center study also found an increased risk of neutropenia with BSA-based dosing [29]. While these data indicate that changes in dosing practices may reduce the risk of breakthrough CMV episodes and drug-associated cytopenias, they will not eliminate it and thus other strategies designed to further reduce CMV reactivation are needed.

Letermovir, which does not cause cytopenia, is now being used by adult and pediatric hematopoietic cell transplant programs for CMV prophylaxis [30–35] and has been recently approved for CMV prophylaxis in adult kidney transplantation [36, 37]. However, there have been no studies in pediatric SOT recipients, and data on adolescent (12–17 years of age) stem cell transplant recipients were only recently published [38]. If proven safe and effective in pediatric SOT and if pharmacokinetic and dosing data are established for younger children, letermovir could provide a viable alternative for prophylaxis in pediatric SOT patients.

The need to consider and study alternative strategies with fewer adverse toxicities for CMV prevention is highlighted by several findings in this study, including the frequency of breakthrough CMV DNAemia, which was temporally associated with higher rates of EBV and adenovirus detection. In addition, episodes of bacteremia were more common among patients who developed neutropenia while on valganciclovir. We also observed a significant association between CMV DNAemia and graft rejection, particularly in liver transplant recipients. However, the absence of detail on the timing of rejection relative to DNAemia precludes drawing any conclusion regarding causality. The association may be bidirectional as CMV may directly or indirectly trigger rejection and conversely, rejection and the associated intensification of immunosuppression may increase the risk of CMV reactivation. However, the association observed is consistent with a large single-center study of pediatric SOT patients receiving universal prophylaxis, which also documented an increased risk of rejection in liver (but not other organ) transplant recipients in association with CMV DNAemia [22]. It has been postulated that the immunomodulatory effects of CMV, even when viral DNA is detected at relatively low levels in the blood, may precipitate graft rejection. Proposed mechanisms include upregulation of adhesion molecules on vascular endothelial cells with the subsequent recruitment of immune cells and an increase in the expression of major histocompatibility complex class II on multiple cell types, which would promote graft rejection [39]. It is also not clear whether the increased rates of other infections, particularly bacteremia, EBV, and adenoviral DNAemia, which were observed more often in patients who experienced breakthrough CMV DNAemia, reflect the immunomodulatory effects of CMV or, rather, that the factors associated with CMV reactivation including neutropenia also increase the risk for bacteremia and trigger EBV and adenovirus reactivation.

Disentangling the factors that contribute to breakthrough CMV DNAemia and its association with graft rejection and other infections is difficult given the retrospective nature of this multicenter study and the lack of data on the precise timing of rejection relative to episodes of CMV DNAemia. We recognize that there are several additional limitations to the study including other potential confounders that may not have been identified and excluded even when controlling for variables in a multivariate analysis. There were no standard protocols for frequency of CMV PCR testing, assays utilized, VGCV dosing, dose adjustments based on perceived toxicities, threshold to start treatment for CMV DNAemia, or immunosuppressive regimens. We did collect data on the immunosuppressive drug prescribed but did not obtain dosing or drug levels. By design, we did not include patients who received no CMV antiviral prophylaxis or in whom a preemptive prophylaxis strategy (eg, introduction of VGCV only when CMV DNA is detected in the blood by frequent PCR monitoring) was used and thus cannot compare the rates of CMV infection based on these different strategies. However, the rates of CMV observed here are consistent with studies that have compared these different approaches [6, 7].

In conclusion, VGCV prophylaxis is associated with a low rate of CMV disease compared to historic controls but does not prevent breakthrough CMV DNAemia, which has potential clinical consequences and poses a challenge to patient management. The greatest burden of these adverse outcomes was observed in liver transplant recipients. These findings suggest that other antiviral agents or strategies need to be studied in pediatric SOT patients, particularly in the liver transplant population. In addition, studies to better understand the pathological link between CMV DNAemia and graft rejection are needed.

## Supplementary Material

ofae353_Supplementary_Data

## Supplementary Data

Supplementary materials are available at *Open Forum Infectious Diseases* online. Consisting of data provided by the authors to benefit the reader, the posted materials are not copyedited and are the sole responsibility of the authors, so questions or comments should be addressed to the corresponding author.

## Notes

***Author contributions.*** M. F., S. D., T. J., and B. C. H. were involved in concept formulation, institutional review board (IRB) submission, data collection, data analysis, and manuscript preparation. F. M. M., K. G. V. D., C. E. B., T. S. S., G. S., W. J. M., T. H. S., L. D.-I., S. Bl., J. B., S. Ba., J. S., M. I. A., Y. S., G. M. M., and J. F. were involved in IRB submission, data collection, and manuscript review.

***Financial support.*** This work was partially supported by Merck & Co.
