# Supplementary material for: Multicenter Analysis of Valganciclovir Prophylaxis in Pediatric Solid Organ Transplant Recipients
Source: Open Forum Infect Dis. 2024 Jul 1;11(7):ofae353. doi: 10.1093/ofid/ofae353 (PMC11229698; doi:10.1093/ofid/ofae353)
Supplement: ofae353_Supplementary_Data [file ofae353_supplementary_data.docx]

**Supplemental Table 1** – Study Sites

| **STUDY SITE** | **Participants**  **n (%)** |
| --- | --- |
| Texas Children’s Hospital Baylor College of Medicine | 247 (33) |
| Boston Children’s Hospital | 168 (22) |
| Lurie Children’s Hospital | 91 (12) |
| Cincinnati Children’s Hospital | 84 (11) |
| Children’s Hospital Colorado | 66 (9) |
| Children’s Hospital at Montefiore | 35 (5) |
| Seattle Children’s Hospital | 30 (4) |
| Nationwide Children’s Hospital | 28 (4) |
| Total | 749 (100) |

**Supplement Table 2** – Valganciclovir Dosing Methods

| **Dosing Method** | **n (%)** |
| --- | --- |
| Weight-Based (15 mg/kg/dose) | 312 (41.7) |
| Body surface area (BSA) (7*BSA*Creatinine Clearance) | 100 (13.4) |
| Adult Dose (900 mg) | 74 (9.9) |
| 500 mg/m^2^ | 43 (5.7) |
| Other/Not documented | 220 (29.3) |
| Total | 749 |

**Supplemental Table 3:** Immunosuppression

|  | **Overall n=749** | **No DNAemia n=618** | **Breakthrough DNAemia n=85** | **Post-PPX DNAemia n=46** | **p** | **p1** | **p2** | **p3** |
| --- | --- | --- | --- | --- | --- | --- | --- | --- |
| **Induction immunosuppression n (%)** |  |  |  |  |  |  |  |  |
| Corticosteroid | 612 (81.7) | 509 (82.4) | 71 (83.5) | 32 (69.6) | 0.09 | 0.8 | 0.03 | 0.06 |
| Anti-thymocyte globulin | 311 (41.5) | 262 (42.4) | 30 (35.3) | 19 (41.3) | 0.5 | 0.2 | 0.9 | 0.5 |
| Basiliximab | 156 (20.8) | 129 (20.9) | 18 (21.2) | 9 (19.6) | 0.9 | 0.9 | 0.8 | 0.8 |
| Alemtuzumab | 71 (9.5) | 69 (11.2) | 2 (2.4) | 0 | 0.003 | 0.01 | 0.02 | 0.3 |
| Rituximab | 11 (1.5) | 10 (1.6) | 1 (1.2) | 0 | 0.7 | 0.8 | 0.4 | 0.5 |
| **Maintenance immunosuppression n (%)** |  |  |  |  |  |  |  |  |
| Tacrolimus | 744 (99.3) | 613 (99.2) | 85 (100.0) | 46 (100.0) | 0.6 | 0.4 | 0.5 | 0.99 |
| Corticosteroid | 597 (79.7) | 490 (79.3) | 71 (83.5) | 36 (78.3) | 0.6 | 0.4 | 0.9 | 0.5 |
| Mycophenolate mofetil | 620 (82.9) | 515 (83.5) | 70 (82.4) | 35 (76.1) | 0.4 | 0.8 | 0.2 | 0.4 |
| Sirolimus | 95 (12.7) | 80 (12.9) | 12 (14.1) | 3 (6.5) | 0.4 | 0.8 | 0.2 | 0.2 |
| Azathioprine | 66 (8.8) | 59 (9.6) | 5 (5.9) | 2 (4.4) | 0.3 | 0.3 | 0.2 | 0.7 |
| Everolimus | 14 (1.2) | 11 (1.8) | 2 (2.4) | 1 (2.2) | 0.9 | 0.7 | 0.8 | 0.9 |
| Cyclosporine | 16 (2.1) | 12 (1.9) | 2 (2.4) | 2 (4.4) | 0.5 | 0.8 | 0.3 | 0.5 |

| **Supplemental Table 4:** Immunosuppression by organ | **Kidney n=294** | **Liver n=227** | **Heart=169** | **p** | **p1** | **p2** | **p3** |
| --- | --- | --- | --- | --- | --- | --- | --- |
| **Induction immunosuppression n (%)** |  |  |  |  |  |  |  |
| Corticosteroid | 234 (79.6) | 201 (88.6) | 123 (72.8) | <0.001 | 0.006 | 0.01 | <0.001 |
| Anti-thymocyte globulin | 140 (47.6) | 4 (1.8) | 131 (77.5) | <0.001 | <0.001 | <0.001 | <0.001 |
| Basiliximab | 81 (27.6) | 46 (20.3) | 10 (5.9) | <0.001 | 0.06 | <0.001 | <0.001 |
| Alemtuzumab | 67 (22.8) | 1 (0.4) | 0 | <0.001 | <0.001 | <0.001 | 0.4 |
| Rituximab | 3 (1.0) | 1 (0.4) | 6 (3.6) | 0.03 | 0.5 | 0.06 | 0.02 |
| **Maintenance immunosuppression n (%)** |  |  |  |  |  |  |  |
| Tacrolimus | 294 (100.0) | 227 (100.0) | 164 (97.0) | <0.001 | >0.99 | 0.003 | 0.009 |
| Corticosteroid | 179 (60.9) | 223 (98.2) | 136 (80.5) | <0.001 | <0.001 | <0.001 | <0.001 |
| Mycophenolate mofetil | 291 (99.0) | 105 (46.3) | 168 (99.4) | <0.001 | <0.001 | 0.6 | <0.001 |
| Sirolimus | 32 (10.9) | 32 (14.1) | 30 (17.8) | 0.1 | 0.3 | 0.04 | 0.3 |
| Azathioprine | 49 (16.7) | 6 (2.6) | 10 (5.9) | <0.001 | <0.001 | 0.001 | 0.1 |
| Everolimus | 1 (0.3) | 0 (0) | 13 (7.7) | <0.001 | 0.4 | <0.001 | <0.001 |
| Cyclosporine | 5 (1.7) | 4 (1.8) | 7 (4.1) | 0.2 | 0.9 | 0.1 | 0.2 |
| p1=Kidney vs Liver, p2=Kidney vs Heart, p3=Liver vs Heart | | | | | | | |

| **Supplemental Table 5:** Infections | **Overall n=749** | **Kidney n=294** | **Liver n=227** | **Heart n=169** | **Lung n=40** | **Other n=19** | **p** |
| --- | --- | --- | --- | --- | --- | --- | --- |
| Any infection | 370 (49.4) | 146 (49.66) | 132 (58.2) | 57 (33.7) | 23 (57.5) | 12 (63.2) | <0.001 |
| Bacterial^1^ | 187 (25.0) | 97 (33.0) | 51 (22.5) | 19 (11.2) | 13 (32.5) | 7 (36.8) | <0.001 |
| Bacteremia | 68 (9.1) | 19 (6.5) | 28 (12.3) | 11 (6.5) | 8 (20.0) | 2 (10.5) | 0.01 |
| Clostridium difficile | 29 (3.9) | 13 (4.4) | 11 (4.9) | 5 (3.0) | 0 | 0 | 0.5 |
| UTI | 97 (13.0) | 73 (24.8) | 12 (5.3) | 3 (1.8) | 5 (12.5) | 4 (21.1) | <0.001 |
| Viral^2^ | 267 (35.7) | 86 (29.3) | 111 (48.9) | 43 (25.4) | 17 (42.5) | 10 (52.6) | <0.001 |
| EBV viremia | 206 (27.5) | 61 (20.8) | 92 (40.5) | 34 (20.1) | 12 (30.0) | 7 (36.8) | <0.001 |
| Adenovirus viremia | 21 (2.8) | 3 (1.0) | 12 (5.3) | 1 (0.6) | 4 (10.0) | 1 (5.3) | 0.001 |
| BK viremia | 21 (2.8) | 21 (7.1) | 0 | 0 | 0 | 0 | <0.001 |
| Norovirus gastroenteritis | 19 (2.5) | 2 (0.7) | 9 (4.0) | 8 (4.7) | 0 | 0 | 0.03 |
| Fungal^3^ | 9 (1.2) | 1 (0.3) | 4 (1.8) | 1 (0.6) | 1 (2.5) | 2 (10.5) | 0.002 |
| Candidemia | 3 (0.4) | 0 | 0 | 0 | 1 (2.5) | 2 (10.5) | <0.001 |
| 1 Other bacterial infections: meningitis (1), pneumonia (4), peritonitis (6), abscess (1), mediastinitis (1) | | | | |  |  |  |
| 2 Other viral infections: HHV6 (13), HSV (2), Enterovirus viremia (2), Sapovirus AGE (2), COVID (1), Parvovirus viremia (4), RSV, Influenza (5), Astrovirus AGE (1), Adenovirus AGE (2), West Nile Meningitis (1), Adeno URI (2) | | | | | | | |
| 3 Other fungal infections: Candida peritonitis (2), Candiduria (1), Pulmonary mold infections (3) | | | | |  |  |  |

| **Supplemental Table 6: Outcome - Hazard Model** | Breakthrough CMV DNAemia | | | |
| --- | --- | --- | --- | --- |
|  | Unadjusted |  | Adjusted |  |
|  | HR, 95% CI | p | HR, 95% CI | p |
| Age, year | 1.0 [0.9-1.1] | 0.1 | 0.9 [0.9-1.1] | 0.5 |
| Organ |  |  |  |  |
| Kidney (reference) |  |  |  |  |
| Liver | 3.8 [2.1-7.0] | <0.001 | 3.5 [1.7-7.0] | <0.001 |
| Heart | 2.3 [1.1-4.6] | 0.01 | 2.4 [1.1-5.2] | 0.02 |
| Lung | 4.4 [1.9-10.5] | 0.001 | 5.3 [2.0-13.7] | 0.001 |
| CMV risk group |  |  |  |  |
| Low (reference) |  |  |  |  |
| Intermediate | 7.6 [1.8-31.7] | 0.006 | 7.4 [1.8-31.4] | 0.006 |
| High | 11.0 [2.7-45.2] | 0.001 | 10.8 [2.6-45.1] | 0.001 |
| Neutropenia on VGCV | 2.2 [1.4-3.4] | <0.001 | 1.5 [0.9-2.5] | 0.09 |
| Lymphopenia on VGCV | 1.9 [1.2-3.0] | 0.004 | 1.3 [0.8-2.1] | 0.4 |
| Toxicity related VGCV modification | 2.6 [1.5-4.4] | <0.001 | 2.3 [1.3-4.2] | 0.005 |
| Bacteremia | 2.4 [1.4-4.2] | 0.002 | 2.1 [1.2-3.8] | 0.01 |
| EBV DNAemia | 1.6 [1.1-2.5] | 0.04 | 1.4 [0.8-2.2] | 0.2 |
| CMV IVIG prophylaxis | 0.9 [0.5-1.7] | 0.8 | 0.8 [0.4-1.7] | 0.5 |
| TMP-SMX | 2.4 [1.2-4.9] | 0.01 | 1.5 [0.7-3.1] | 0.3 |

| **Supplemental Table 7:** | Rejection in 1 year |  |  |  |
| --- | --- | --- | --- | --- |
|  | Unadjusted |  | Adjusted |  |
|  | OR, 95% CI | p | OR, 95% CI | p |
| Organ (Kidney =reference) |  |  |  |  |
| Liver | 2.6 [1.7-4.1] | <0.001 | 2.4 [1.5-3.8] | <0.001 |
| Heart | 3.0 [1.8-4.8] | <0.001 | 2.9 [1.8-4.6] | <0.001 |
| Lung | 0.9 [0.3-2.5] | 0.9 | 0.8 [0.3-2.2] | 0.7 |
| Breakthrough CMV DNAemia | 2.1 [1.3-3.4] | 0.003 | 1.9 [1.1-3.1] | 0.01 |
| Breakthrough CMV by transplant organ: | | | | |
| Kidney | 0.5 [0.1-3.8] | 0.5 |  |  |
| Liver | 2.9 [1.4-5.8] | 0.004 |  |  |
| Heart | 1.6 [0.6-4.4] | 0.3 |  |  |
| Lung | 2.7 [0.4-19.2] | 0.3 |  |  |
